# Single‐Cell and Multiomics Characterization of p21 in Cancer Progression and Therapeutic Sensitivity

**DOI:** 10.1155/humu/5598948

**Published:** 2026-07-06

**Authors:** Wenyang Zhang, Qinglong Ma, Honghui Zhao, Wenrun Li, Zidong Feng, Linghui Zhong, Yaru Ren, Lin Chang, Yonghong Li, Lei Shi

**Affiliations:** ^1^ RNA Oncology Group, School of Public Health, Lanzhou University, Lanzhou, China, lzu.edu.cn; ^2^ College of Life Science, Lanzhou University, Lanzhou, China, lzu.edu.cn; ^3^ Cuiying Honors College, Lanzhou University, Lanzhou, China, lzu.edu.cn; ^4^ NHC Key Laboratory of Diagnosis and Therapy of Gastrointestinal Tumor, Gansu Provincial Hospital, Lanzhou, China, gsyy.cn; ^5^ College of Veterinary Medicine, Lanzhou University, Lanzhou, China, lzu.edu.cn; ^6^ Cancer Research UK Manchester Institute, The University of Manchester, Manchester, UK, manchester.ac.uk

**Keywords:** *CDKN1A*/p21, cell cycle, prognostic biomarker, scRNA-seq, tumor microenvironment

## Abstract

**Introduction:**

*CDKN1A* (encoding p21) is a canonical regulator of cell cycle arrest and genomic stability. However, its functional spectrum within the tumor microenvironment (TME), especially at single‐cell resolution, remains insufficiently defined.

**Methods:**

We performed an integrative multiomics analysis combining bulk transcriptomics, single‐cell RNA sequencing (scRNA‐seq), and functional assays across multiple cancer types. Publicly available datasets were used to assess *CDKN1A* expression, immune infiltration, and signaling pathways. Functional validation was conducted upon silencing or overexpression of p21 in several cancer cell lines.

**Results:**

Pancancer analysis revealed that *CDKN1A* expression is frequently dysregulated across multiple tumor types and is associated with patient prognosis. ScRNA‐seq further demonstrated that *CDKN1A* was preferentially enriched in nonmalignant epithelial cells and varies along an inferred malignant epithelial transcriptional continuum. Computational cell–cell communication analysis suggested inferred ligand‐receptor associations involving extracellular matrix‐related and immune‐associated signaling pathways. Functionally, p21 overexpression suppressed tumor cell proliferation, induced senescence and apoptosis, and reduced migratory capacity across multiple cancer cell lines. In vitro drug‐sensitivity assays further showed that p21 overexpression was associated with enhanced sensitivity to targeted therapy and chemotherapy agents, as evidenced by reduced IC50 values. In addition, p21 regulated immune‐related molecules, including *ICOS*, *TGFB1*, and *PDCD1*, and coculture experiments revealed that macrophage polarization differentially modulates p21‐mediated tumor cell inhibition, with M1 macrophages enhancing and M2 macrophages attenuating this effect.

**Discussion:**

By delineating its function at single‐cell resolution, we identify *CDKN1A* as a prognostic biomarker and a potential modulator of tumor‐cell drug sensitivity that requires further clinical validation. These findings expand the conventional view of p21 from a cell cycle inhibitor to a context‐dependent regulator of tumor immune interactions and therapeutic sensitivity‐associated phenotypes.

## 1. Introduction

Cancer has become a major global public health challenge, driven by complex genetic and epigenetic alterations that promote tumor initiation and progression [1]. Tumorigenesis is a dynamic, multilevel process involving genomic instability, epigenetic modifications, and dysregulated cell signaling [2]. Among the hallmarks of cancer, dysregulation of the cell cycle is a central feature, enabling tumor cells to evade growth suppression and acquire malignant properties [3]. The cell‐dependent kinase inhibitor 1A (*CDKN1A*) gene, coding p21 protein, is a critical regulator of the cell cycle that functions mainly by blocking cyclin‐CDK complexes, leading to G1 phase arrest [4]. As a well‐known downstream effector of p53, *CDKN1A*/p21 is responsible for different molecular biological functions such as differentiation, cell migration/invasion, apoptosis, DNA repair and stem cell reprogramming [5, 6]. Emerging studies have demonstrated that p21 can serve as both a tumor suppressor and an oncogene, largely depending on factors such as TME, cell types, subcellular localization, and histone modification [7, 8].

Cancer heterogeneity refers to the diversity of cancer cells within a single tumor (intratumor heterogeneity) or between tumors (intertumor heterogeneity) [9, 10]. This heterogeneity arises from diverse genetic, epigenetic, and phenotypic variations among tumor cells [11]. This heterogeneity poses significant challenges for diagnosis, prognosis, and treatment, necessitating advanced technologies to dissect the molecular underpinnings of cancer at high resolution. To unravel this complexity, scRNA‐seq has emerged as a transformative technology, enabling high‐resolution analysis of gene expression at the individual cell level [12, 13]. In contrast to bulk RNA sequencing, which captures average expression across mixed cell populations, scRNA‐seq allows for the identification of rare cell types and reveals dynamic cellular states within the TME [14]. Recent studies have hinted at its potential involvement in immune cell infiltration and mismatch repair (MMR) pathways, suggesting broader implications for immunotherapy [15, 16]. However, a systematic investigation of *CDKN1A*′s roles at single‐cell resolution is lacking, leaving critical gaps in our understanding of its contributions to tumor biology and treatment resistance.

In this study, we employ a multidisciplinary approach, combining functional assays, bioinformatics analyses, and scRNA‐seq, to elucidate the diverse roles of *CDKN1A* across multiple cancer types. We explore its expression patterns, prognostic significance, and associations with immune infiltration while also investigating its impact on chemotherapeutic sensitivity. Our findings aim to redefine *CDKN1A* not only as a cell cycle regulator but also as a key modulator of the TME and a potential biomarker for precision oncology. By integrating single‐cell genomics with experimental validation, we provide a comprehensive framework for understanding the biological and clinical relevance of *CDKN1A*, paving the way for novel therapeutic strategies.

## 2. Materials and Methods

### 2.1. Cell Culture

The lung cancer cell lines H1299 (Cat no. CL‐0165, RRID CVCL_0060), A549 (Cat no. CL‐0016, RRID CVCL_0574), and the breast cancer cell line BT474 (Cat no. CL‐0040, RRID CVCL_0179) were cultured in RPMI‐1640 medium. The breast cancer cell line MCF‐7 (Cat no. CL‐0149, RRID CVCL_0031) as well as the pancreatic cancer cell lines Mia (Cat no. CL‐0627, RRID CVCL_0428) and PDC0034 cells were cultured in Dulbecco′s modified Eagle′s medium (DMEM) in supplemented with 10% Fetal Bovine Serum (FBS) and 100 units/mL penicillin/streptomycin. THP‐1 (Cat no. CL‐0233, RRID CVCL_0006) cells were cultured in PRMI‐1640 in supplemented with 10% FBS, 100 units/mL penicillin/streptomycin, and 0.05 mM *β*‐mercaptoethanol. PDC0034 was kindly provided by Professor Zuoyi Jiao (Lanzhou University), and all other cell lines were purchased from Wuhan Pricella Biotechnology Co. Ltd. and maintained at 37°C in a humidified atmosphere containing 5% CO_2_. Cell lines used in this study were authenticated by short tandem repeat (STR) profiling, which confirmed a 100% match with their reference profiles in the ATCC databases. All cell lines were routinely tested and confirmed to be negative for mycoplasma contamination throughout the experiments.

### 2.2. Reagents and Antibodies

Cisplatin (HY‐17394) and Trametinib (HY‐10999) were purchased from MedChemExpress (MCE). Antibodies including p21 (rabbit polyclonal, Cat. No. 10355‐1‐AP, Proteintech, 1:2000), GCNF (rabbit polyclonal, Cat. No. 12712‐1‐AP, Proteintech, 1:1000), CCND3 (rabbit polyclonal, Cat. No. 26755‐1‐AP, Proteintech, 1:1000), GAPDH (mouse monoclonal, Cat. No. 60004‐1‐Ig, Proteintech, 1:100000), *α*‐Tubulin (mouse monoclonal, Cat. No. 66031‐1‐Ig, Proteintech, 1:50000), HRP‐conjugated goat anti‐mouse IgG (H + L) (Cat. No. SA00001‐1, Proteintech, 1:10000), HRP‐conjugated goat anti‐rabbit IgG (H + L) (Cat. No. SA00001‐2, Proteintech, 1:10000), and CD137 (rabbit monoclonal, Cat No. A22167, ABclonal, 1:1000) were applied for the Western blot.

### 2.3. siRNA and Plasmid Transfection

siRNA (genOFFTM st‐h *CDKN1A*_001, Cat No. stB0001992A, GATGGAACTTCGACTTTGT; target gene ID: ENSG00000124762) was designed and purchased from Guangzhou RiboBio Co. Ltd and transfected at a concentration of 50 nM using HiPerFect (Qiagen) for 48 h, following the manufacturer′s instructions. Plasmids (pCDH‐Flag‐MYC, Addgene, catalogue number 102626; p21, Origene, catalogue number RC201765) were transiently transfected into cells for 48 h with lipofectamine 2000 (Thermo Fisher Scientific) reagent according to the manufacturer′s protocol.

### 2.4. RNA Extraction and RT‐qPCR

Total RNA was isolated using the M5 Total RNA Extraction Reagent (Mei5bio). cDNA was generated from 1 *μ*g of total RNA per sample using the M5 Sprint qPCR RT kit (Mei5bio). Quantitative real‐time PCR (RT‐qPCR) was performed by using a Light Cycler 480 (Roche) and the Fast SYBR Green Master Mix (Mei5bio). The RT‐qPCR primer sequences are shown in Table 1.

**Table 1:** RT‐qPCR primers.

Gene	Primer	Sequences
*PPIC*	Fwd	CTTTATCACCTTGACCAAGCC
	Rev	ACACACACACACACACAC
*TNFRSF9*	Fwd	GTTGCTCTTCCTGCTGTTC
	Rev	CACATCCTCCTTCTTCTTCTTC
*BHLHE40*	Fwd	TTCTCCCTTGCCAGCTCATC
	Rev	ACACACACACACACACACAC
*ADAM19*	Fwd	AACCCTCAAACCACCACAC
	Rev	TGCTCACCGTAATCAGTCC
*NR6A1*	Fwd	TATTTGCCCTGCTTTGCC
	Rev	ATCACCTCCATCCCTTCATC
*RBPJ*	Fwd	TTCCTGGACAATCATTAGCAC
	Rev	TCTACATCCCCAAACCACAC
*PDCD6*	Fwd	CGAAACCCCATCTCAAAAAAAC
	Rev	ACAACACAACTATGAACCTTCC
*PTPMT1*	Fwd	TGTAAGAGCCATCGCCAAG
	Rev	TGAAATGACAAAAGTCCCATCC
*MET*	Fwd	TACCCCAGCCCAAACCATTTC
	Rev	ATGCCACTGTAAAGTTCCTTCC
*CCND3*	Fwd	ACCACACCACATCTAAGCC
	Rev	CAATTCTGTCCCATCAGCC
*EPCEM*	Fwd	TGACAGTAAATGGGGAACAAC
	Rev	TCACCACAACCACAATAACAG
*PMS2*	Fwd	TGGATGTGGGGTAGAAGAAG
	Rev	GTGGCAGGTAGAAATGGTG
*MSH2*	Fwd	AAGGCTTCTCCTGGCAATC
	Rev	TCCACATACCCAACTCCAAC
*CDKN1A*	Fwd	TCACTGTCTTGTACCCTTGTGC
	Rev	GGCGTTTGGAGTGGTAGAAA
*β-Actin*	Fwd	TGACATTAAGGAGAAGCTGTGCTAC
	Rev	GAGTTGAAGGTAGTTTCGTGGATG
*ICOS*	Fwd	CCTCTCAAAACAAACACCCTC
	Rev	ATTCAGTACCCCTGGCATC
*TGFB1*	Fwd	CTGCACTATTCCTTTGCCC
	Rev	TCTTCTTCACTATCCCCCAC
*PDCD1*	Fwd	TGACAGAGAGAAGGGCAGAAG
	Rev	TCCACAGAGAACACAGGCAC
*CD86*	Fwd	CTTCCTGCTCTCTGCTAACTTC
	Rev	GCTGATGGAAACGTCGTACA
*HLA-DR*	Fwd	ATTTTTCTGATTGGCCAAAGAGTAATT
	Rev	AAAAGAAAAGAGAATGTGGGGTGTAA
*CD206*	Fwd	CTCTGTTCAGCTATTGGACGC
	Rev	TGGCACTCCCAAACATAATTTGA
*CD263*	Fwd	GGACATGAGTCCCATCTTTCAC
	Rev	AGCTCCACTCTGCCCTCACAC
*ICOS*	Fwd	CCTCTCAAAACAAACACCCTC
	Rev	ATTCAGTACCCCTGGCATC

### 2.5. Protein Extraction and Western Blot

Total protein was extracted by homogenizing cells in 1 × RIPA buffer (Boster) supplemented with protease inhibitors and phosphatase inhibitors for 20 min, followed by centrifugation at 12,000 rpm for 20 min at 4°C. Western blot was performed as previously described [17].

### 2.6. Immunofluorescence Staining

Cells were grown on coverslips for 48 h and fixed with 4% PFA at room temperature for 10 min, permeabilized with 0.2% Triton X‐100/PBS for 5 min at room temperature. Coverslips were then incubated with anti‐p21 primary antibody (rabbit polyclonal, Cat. No. 10355‐1‐AP, Proteintech, 1:100) and CoraLite594‐conjugated goat anti‐rabbit IgG(H + L) (Cat. No. SA00013‐4, Proteintech, 1:100) secondary antibody. Digital photographs were acquired with a ZEISS LSM910 Airscan confocal microscope.

### 2.7. CCK8 Assay

Cells transfected with plasmids or siRNA were seeded at 5000 cells per well in 96‐well plates. After 48 h, CCK‐8 detection reagent (APExBIO, K1018) was added and incubated for 4 h. Optical density (OD) values were measured at 450 nm using a microplate reader.

### 2.8. 5‐Ethynyl‐2 ^′^‐Deoxyuridine Assay (EdU)

Cells were incubated with freshly prepared EdU buffer (Ribobio) for 2 h. After three washes with PBS, cells were fixed and stained with Apollo reaction cocktail for 30 min, followed by Hoechst 33342 staining. Fluorescence microscopy was used to visualize EdU‐positive cells.

### 2.9. Colony Formation Assay

2 × 10^3^ Pretreated cells were seeded in each well of six‐well plates for 14 Days. Colonies were subsequently washed with PBS, fixed with cold methanol, stained with 0.05% crystal violet (BKMAM), and counted using the ImageJ software.

### 2.10. Annexin‐V Assay

H1299, A549, MCF‐7, BT474, Mia, and PDC0034 cells were transfected with p21 plasmid in 6‐well plates for 48 h. Apoptosis was assessed using the Annexin V‐FITC/PI Apoptosis kit (Elabscience) according to the manufacturer′s instructions. The percentage of apoptotic cells was analyzed using a flow cytometer (BD Accuri C6).

### 2.11. Wound Healing Assay

To stop cell proliferation, mitomycin C (10 ng/mL) was applied 2 h before wound generation. After removing the Culture‐insert (IBIDI), cells were washed twice with PBS solution to eliminate mitomycin C and detached cells. The cells were then allowed to migrate and close the wound for 24–48 h, after which images were captured and evaluated microscopically.

### 2.12. *β*‐Galactosidase Staining

Cell senescence was assessed using *β*‐galactosidase (SA‐*β*‐gal) staining kits (Beyotime Biotechnology). Briefly, the cells were washed once with PBS and fixed with *β*‐galactosidase staining fixative solution for 15 min at room temperature. After three times with PBS, the cells were then incubated with SA‐*β*‐gal staining solution at 37°C overnight, followed by observation under an optical microscope.

### 2.13. THP‐1 Differentiation, M1/M2 Polarization, and Coculture

THP‐1 cells were seeded in six‐well plates and cultured for 24 h, followed by treatment with PMA (MCE, HY‐18739) at a final concentration of 50 ng/mL for an additional 24 h to induce differentiation into macrophages. The PMA‐containing medium was then removed, and cells were gently washed once with PBS, and fresh medium was added for a 24 h resting period.

For M1 polarization, differentiated macrophages were stimulated with LPS (Thermo Fisher, 00‐4976‐93; 100 ng/mL) and IFN‐*γ* (Thermo Fisher, 300‐02; 20 ng/mL) for 24 h. Successful polarization was confirmed by assessing mRNA expression levels of *CD86* and *HLA-DR* using RT‐qPCR. Conditioned medium was then collected and applied to p21 plasmid‐transfected cancer cells for 48 h prior to subsequent functional assays.

For M2 polarization, macrophages were treated with IL‐4 (Thermo Fisher, 300‐02; 20 ng/mL) for 24 h. M2 polarization was validated by measuring the expression of *CD206* and *CD163* via RT‐qPCR. The conditioned medium was subsequently collected and used to coculture with p21 plasmid‐transfected cells for 48 h for downstream functional analyses.

### 2.14. *CDKN1A* Expression in Diverse Cancer Types

The expression levels of *CDKN1A* across various cancer types were examined utilizing the public database UCSC Xena [18] and TIMER [19], respectively. Transcriptomic RNA‐seq data, DNA methylation data, and phenotype data for 33 cancers were obtained from the TCGA database. These cancers include adrenocortical carcinoma (ACC), Bladder urothelial carcinoma (BLCA), breast invasive carcinoma (BRCA), colon adenocarcinoma (COAD), lymphoid neoplasm diffuse large B‐cell lymphoma (DLBC), esophageal carcinoma (ESCA), glioblastoma multiforme (GBM), head and neck squamous cell carcinoma (HNSC), kidney chromophobe (KICH), kidney renal clear cell carcinoma (KIRC), kidney renal papillary cell carcinoma (KIRP), acute myeloid leukemia (LAML), brain lower grade glioma (LGG), liver hepatocellular carcinoma (LIHC), lung adenocarcinoma (LUAD), lung squamous cell carcinoma (LUSC), ovarian serous cystadenocarcinoma (OV), pancreatic adenocarcinoma (PAAD), prostate adenocarcinoma (PRAD), rectum adenocarcinoma (READ), skin cutaneous melanoma (SKCM), stomach adenocarcinoma (STAD), testicular germ cell tumors (TGCT), thyroid carcinoma (THCA), thymoma (THYM), Uterine corpus endometrial carcinoma (UCEC), cholangiocarcinoma (CHOL), cervical squamous cell carcinoma and endocervical adenocarcinoma (CESC), mesothelioma (MESO), uveal melanoma (UVM), pheochromocytoma and paraganglioma (PCPG), sarcoma (SARC), uterine carcinosarcoma (UCS).

Furthermore, we utilized the HPA database (https://www.proteinatlas.org/) to examine the differential expression of *CDKN1A* at the protein level [20].

### 2.15. Immunohistochemistry (IHC)

Formalin‐fixed, paraffin‐embedded (FFPE) tissue blocks were obtained from metastatic patients at the Second Hospital of Lanzhou University with informed consent. Tissues were fixed in 4% paraformaldehyde and embedded in paraffin for histological and immunohistochemical analyses. Immunohistochemical staining was performed using a Leica BOND‐MAX automated staining system (Leica Microsystems). Signal detection was conducted using 3,3 ^′^‐diaminobenzidine (DAB; Bond Polymer Refine Detection Kit, Leica Microsystems), followed by hematoxylin counterstaining. Images were acquired using a digital slide scanner (Servicebio Biosystems) and independently evaluated by a pathologist using the H‐score method.

### 2.16. scRNA‐Seq Data Collection and Preprocessing

For specific cancer analyses, we obtained data of pancreatic cancer samples from GSE205013 and GSE155698 of NCBI GEO database [21, 22], the lung cancer samples from Code Oceanic capsule 10.24433/CO.0121060.v1, HRA001130 on Beijing Institute of Genomics (BIG), and GSE149813 of NCBI GEO database [23–25]. Breast cancer samples were retrieved from GSE176078 [26]. All datasets were processed using the Seurat R package [27]. Low‐quality cells were filtered based on standard quality control metrics (mitochondrial content < 20% and feature counts between 200 and 7500). To address batch effects across datasets, the “Harmony” algorithm was implemented to integrate data across samples [28]. Integration was performed on the Top 30 principal components (PCs) derived from the Top 2000 variable features, with “orig.ident” specified as the batch variable to align gene expression profiles across samples while preserving biological variability. Following integration, a K‐nearest neighbor (KNN) graph was constructed using the aligned embeddings. Cell clusters were identified using the FindClusters function with a resolution parameter of 0.2, and the dimensionality was further reduced using the RunUMAP function (dims = 1 : 15) to generate two‐dimensional visualizations.

### 2.17. Functional Characterization, Trajectory Inference, and Interaction Analysis

To analyze the epithelial component of the cancer samples and to infer large‐scale copy number variation (CNV) profiles, the R package “inferCNV” was employed [29]. Briefly, epithelial cells were used as observation cells, whereas nonepithelial cells annotated by canonical immune and stromal markers, including PTPRC‐positive immune cells and COL1A1/DCN‐positive fibroblast‐like stromal cells, were used as reference populations to estimate the baseline expression profile. The inferCNV object was generated from raw UMI counts using chromosomal gene‐order information, and the analysis was performed with standard smoothing and denoising procedures (*c* *u* *t* *o* *f* *f* = 0.1, cluster_by_groups = TRUE, denoise = TRUE, HMM = TRUE). Epithelial cells showing broad chromosome‐arm‐level CNV gains or losses and clustering within high‐CNV inferCNV subgroups were classified as malignant epithelial cells, whereas epithelial cells lacking broad CNV deviations and displaying reference‐like CNV profiles were classified as nonmalignant epithelial cells. For downstream analyses, malignant epithelial cells were then stratified based on *CDKN1A* expression. Specifically, *CDKN1A* expression values were extracted at the single‐cell level, and cells were dichotomized into “high” and “low” groups using the median expression as the primary cutoff. To assess the robustness of this stratification and minimize potential threshold‐dependent bias, alternative quantile‐based grouping strategies were additionally applied, including upper versus lower quartile (Q75 vs. Q25) and 80th versus 20th percentile cutoffs. These alternative groupings were used to evaluate the stability of downstream analytical results. Pathway activity inference was performed using the PROGENy algorithm [30]. Pathway scores were computed from gene expression data and integrated into the Seurat object as a separate assay. Trajectory analysis was performed using Monocle3 to infer transcriptional continua within malignant epithelial cells [31–33]. Briefly, raw UMI count matrices from malignant epithelial subsets were used to construct cell_data_set objects, followed by preprocessing, dimensionality reduction, clustering, and principal graph learning. To avoid potential bias introduced by predefined marker‐based rooting, the trajectory root was determined in a data‐driven manner by selecting representative principal graph nodes within each partition based on cell density distribution, rather than anchoring to *CDKN1A* expression [34]. To independently assess the relationship between cellular differentiation states and *CDKN1A* expression, we employed CytoTRACE [35], a computational framework that predicts differentiation states based on transcriptional diversity (gene count signature). Raw UMI counts were used as input to calculate potency scores, which were then correlated with *CDKN1A* expression to evaluate transcriptional state heterogeneity.

For each gene, single‐cell expression values were grouped into 50 equal pseudotime bins, and the mean expression within each bin was calculated. Smoothed expression trends were plotted using ggplot2 in R (Version 4.4) [36]. Spearman′s rank correlation coefficient (*r*) was used to quantify the monotonic association between gene expression and pseudotime. Furthermore, to explore putative intercellular communication within the tumor microenvironment, the R package “CellChat” was utilized to infer ligand‐receptor interaction probabilities between different cell populations [37]. These CellChat results were interpreted as computationally inferred ligand‐receptor interaction networks rather than direct experimental evidence of functional cell–cell communication.

### 2.18. Tumor Immune Infiltration and Methylation Analysis

The relationship between *CDKN1A* expression and tumor immune infiltration was analyzed using TIMER and TISIDB [38, 39].

### 2.19. Gene Signature Analysis

The gene expression files for different cancer types were acquired from the web‐based tool Broad GDAC Firehose [40]. The data were divided into two subclasses (*CDKN1A* low and *CDKN1A* high) based on the mean level of *CDKN1A*. Gene set enrichment analysis (GSEA) was then used to determine signature enrichment in different cancer types [41]. A bubble diagram was generated using the R package ggplot2 to visualize the general biological function in BLCA, COAD, LUAD, and LUSC. We further utilized the CancerSEA database to analyze the correlation of *CDKN1A* with different functional states in 14 cancers [42].

### 2.20. Statistical Analysis

All data are presented as the mean value ± SD (*n* = 3), unless otherwise indicated differently. The significances were calculated by two paired *t*‐tests.  ^∗^ *p* < 0.05,  ^∗∗^ *p* < 0.01, and  ^∗∗∗^ *p* < 0.001 were defined as statistically significant. Spearman′s correlation was calculated using the GraphPad Prism package (GraphPad Software Inc.)

## 3. Results

### 3.1. The *CDKN1A* Expression in Cancers

**Figure 1:**
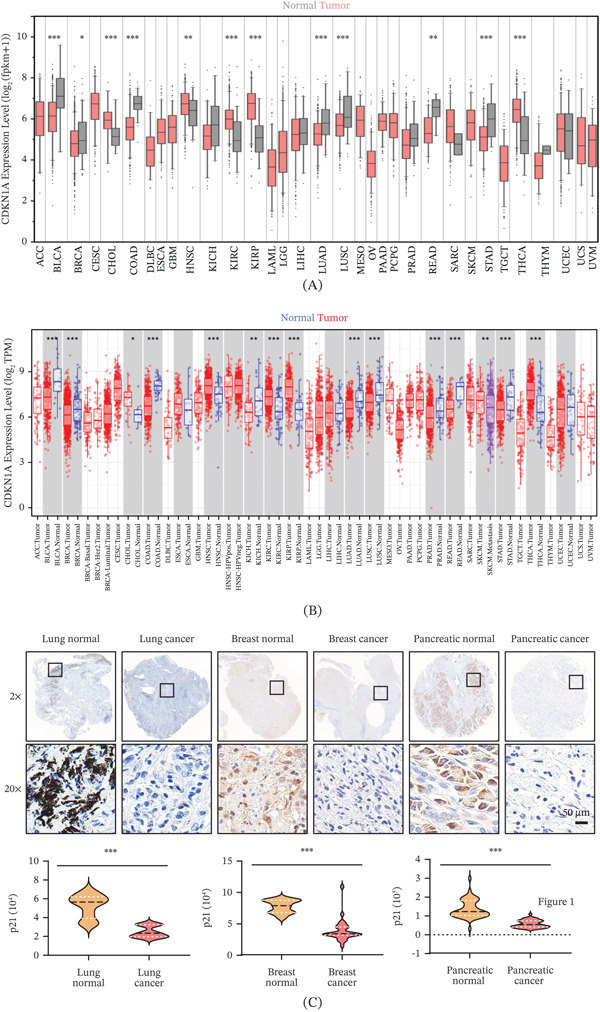
*CDKN1A* level in pancancer. *CDKN1A* mRNA level from the (A) cBioPortal database and (B) TIMER database. (C) IHC staining showing the low expressed p21 protein level in lung cancer, breast cancer, and pancreatic cancer. *Note:* Lung normal *n* = 9, lung cancer *n* = 9; breast normal *n* = 11, breast cancer *n* = 27; pancreatic normal *n* = 48, pancreatic cancer *n* = 48.  ^∗^ *p* < 0.05,  ^∗∗^ *p* < 0.01,  ^∗∗∗^ *p* < 0.001 by two‐tailed Student′s *t*‐test.

To gain better insights into *CDKN1A*, we first examined its expression in various cancer types. We downloaded the raw TCGA data from UCSC Xena and processed the expression value of *CDKN1A* among cancers via the R package. *CDKN1A* was lowly expressed in BLCA, BRCA, COAD, KICH, LUAD, LUSC, READ, and STAD compared with normal samples, whereas it is highly expressed in CHOL, HNSC, KIRC, KIRP, and THCA compared with normal cohorts (Figure 1A). We found similar results via the TIMER database (Figure 1B).

To further confirm these findings at the protein level, we performed immunohistochemical (IHC) staining. Consistently, p21 expression was reduced in lung and breast cancer tissues. In pancreatic cancer, although TCGA data were inconclusive due to the lack of normal controls, IHC analysis revealed that p21 was also downregulated in tumor tissues compared with normal samples (Figure 1C). In addition, data from the Human Protein Atlas demonstrated decreased p21 protein expression in colon, breast, and prostate cancers, consistent with the mRNA expression patterns observed in these tumor types (Figure S1A).

To assess the prognostic value of *CDKN1A*, we analyzed its expression across different tumor stages. Compared with normal samples, *CDKN1A* level was lower in the late stages of BLCA, BRCA, COAD, LUAD, LUSC, KICH, and READ (Figure S1B, S1C, S1D, S1E, S1F, S1G, S1H), whereas the opposite trend was observed in CHOL, HNSC, KIRC, KIRP, and THCA (Figure S1I, S1J, S1K, S1L, S1M), which was consistent with the *CDKN1A* mRNA levels in pancancer (Figure 1A,B). In summary, these results indicate *CDKN1A* may function as a potential prognostic biomarker in cancer.

### 3.2. Gene Signature Analysis

To elucidate the molecular pathways associated with *CDKN1A* expression, we conducted gene signature analysis using TCGA datasets. Our results revealed consistent enrichment of several key oncogenic and immune‐related pathways across four cancer types, including the p53 signaling pathway, apoptosis, TGF‐*β* signaling, IL6‐JAK‐STAT3 signaling, KRAS signaling, G2/M checkpoint, and MYC targets (Figure S2A). To experimentally validate the computational findings, we performed Western blot and immunofluorescence staining, confirming that enforced MYC suppressed p21 in both lung, breast, and pancreatic cancer cells (Figure S2B, S2C), further supporting the interplay between p21 and MYC signaling. Gene Ontology (GO) enrichment analyses suggested that *CDKN1A* was associated with DNA repair, Mitotic Spindle, Catalytic Activity in LUSC (Figure S2D), DNA repair, cell killing, and DNA helicase in COAD (Figure S2E), histone binding and ribosome in BLCA (Figure S2F), RNA splicing, RNA localization, RNA exportation, and cytokine binding in LUAD (Figure S2G).

We next investigated the correlation between *CDKN1A* expression and the functional status across multiple cancer types using the CancerSEA database [42]. *CDKN1A* was significantly associated with several key cellular activities, including cell‐dependent functions, hypoxia, inflammation, and metastasis (Figure S3A). Specifically, in lung cancer, *CDKN1A* expression was notably associated with apoptosis, cell cycle progression, cell differentiation, DNA damage response, epithelial‐mesenchymal transition (EMT), inflammatory signaling, invasion, and metastatic potential (Supplementary Figure S3B, S3C).

Collectively, these findings highlight the diverse and context‐dependent roles of *CDKN1A* in cancer, suggesting its involvement in multiple biological processes, including cell‐cycle regulation, apoptosis, transcriptional control, and immune modulation.

### 3.3. *CDKN1A* Regulates Cell Proliferation

**Figure 2:**
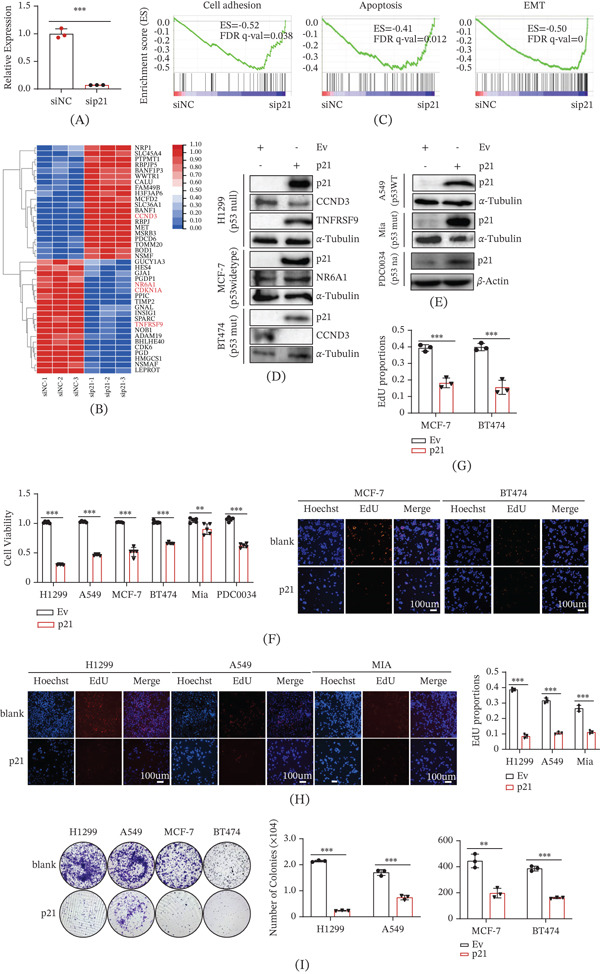
*CDKN1A* regulates cell proliferation. (A) RT‐qPCR result showing *CDKN1A* expression upon siRNA transfection in H1299 cells. (B) Heatmap showing the differentially expressed genes upon p21 silencing in H1299. (C) GSEA analysis of the RNA‐seq indicating p21 affects cell adhesion, apoptosis, and EMT. (D) Western blot revealing p21 regulates downstream genes. (E) p21 overexpression inhibits cell proliferation via (F) CCK8 assay, (G, H) EdU assay, and (I) colony formation in different cell lines. *Note:* F, *n* = 5; G–I, *n* = 3. Scale bar, 100 *μ*m.  ^∗∗∗^ *p* < 0.001,  ^∗∗^ *p* < 0.01 by two‐tailed Student′s *t-*test.

Next, we performed a series of cellular experiments to elucidate the biological functions of *CDKN1A*/p21 in regulating cancer cell proliferation. RNA‐seq was conducted following siRNA‐mediated knockdown of p21 in H1299 to identify the downstream gene expression alterations (Figure 2A). A total of 143 genes were upregulated and 813 genes were downregulated (Figure 2B). GSEA analysis revealed that p21 affects cell adhesion, apoptosis, and EMT (Figure 2C).

To validate these transcriptomic findings, we selected and confirmed the Top 10 most differentially expressed genes at both the mRNA and protein levels. Notably, enforced expression of p21 led to the downregulation of *CCND3* and the upregulation of *TNFRSF9* and *NR6A1* across multiple cancer cell lines, including H1299, A549, MCF‐7, and BT474 (Figure S4; Figure 2D).

**Figure 3:**
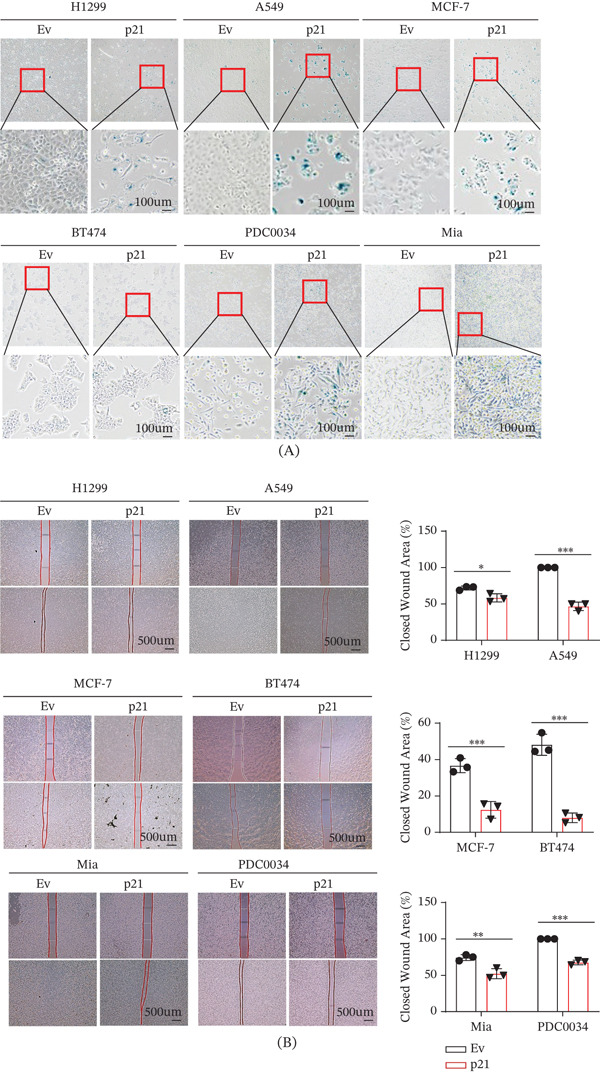
p21 inhibits tumorigenesis. (A) SA‐*β*‐gal staining assay indicating overexpression of p21 promotes cell senescence in different cell lines. Scale bar, 100 *μ*m. (B) Enforced p21 prevents wound closure in different cell lines. *Note:*  ^∗^ *p* < 0.05,  ^∗∗^ *p* < 0.01,  ^∗∗∗^ *p* < 0.001 by two‐tailed Student′s *t-*test.

Functional assays revealed that p21 overexpression dramatically reduces cell proliferation and clonogenic ability in lung cancer cells (H1299 and A549), breast cancer cells (MCF‐7 and BT474), and pancreatic cancer cells (Mia) (Figure 2D–I). To further assess the functional consequences of p21 upregulation in cancer cells, we performed senescence, apoptosis, and wound‐healing assays across multiple tumor types. SA‐*β*‐gal staining demonstrated a marked increase in blue‐stained cells following p21 overexpression, indicating a robust induction of cellular senescence in lung (H1299, A549), breast (MCF‐7, BT474), and pancreatic cancer (PDC0034, Mia) cell lines (Figure 3A). Consistent with this finding, Annexin V/PI staining confirmed a significant elevation in apoptotic cell populations upon p21 induction, suggesting that p21 promoted both growth arrest and programmed cell death (Figure S5A, S5B, S5C, S5D, S5E, S5F, S5G). In addition, the wound‐healing assay revealed that enforced p21 expression substantially impaired the migratory capacity of cancer cells (Figure 3B). Quantifications of wound closure over time showed that p21‐overexpressing cells exhibited delayed or incomplete wound closure compared to controls, underscoring the inhibitory effect of p21 on cell motility. Together, these results indicate that p21 not only triggers senescence and apoptosis but also limits the migratory and invasive potential of tumor cells, reinforcing its role as a tumor suppressor across multiple cancer contexts.

### 3.4. Molecular Landscape of *CDKN1A* at Single Cell Level

**Figure 4:**
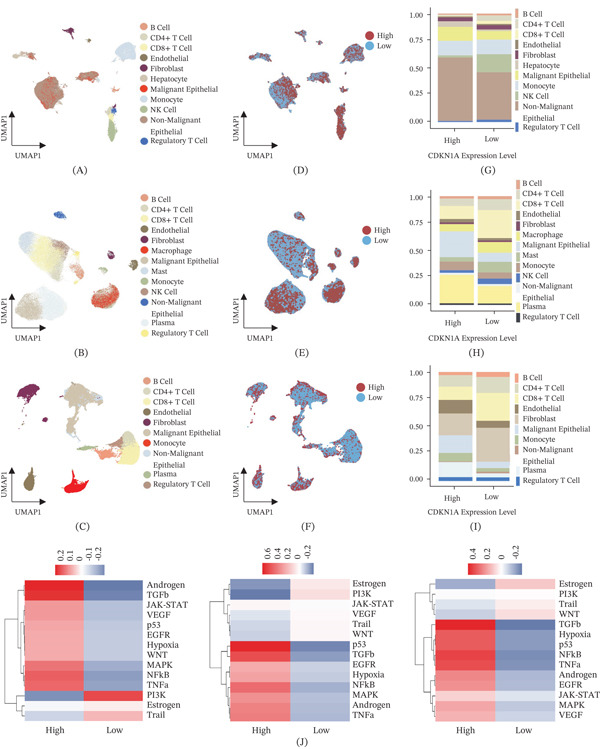
scRNA‐seq profiling revealing the relevance of *CDKN1A* with cell types in pancreatic, lung, and breast cancer. UMAP plot showing the distributions of the cells in (A) pancreatic cancer, (B) lung cancer, and (C) breast cancer. Expression levels of *CDKN1A* in different cell types in (D) pancreatic cancer, (E) lung cancer, and (F) breast cancer. Relative proportions of different cell types with high or low *CDKN1A* expression in (G) pancreatic cancer, (H) lung cancer, and (I) breast cancer. (J) Pathway activities inferred from different gene levels in pancreatic cancer (Left), lung cancer (Middle), and breast cancer (Right).

To further investigate the potential role of *CDKN1A* within the tumor microenvironment, we performed scRNA‐seq analysis focusing on pancreatic, lung and breast cancers, all of which previously exhibited significant associations with *CDKN1A* expression in bulk transcriptomic data and in our cellular experiments. Given that adenocarcinoma originate from epithelial cells [43], we applied inferCNV analysis to epithelial cell subsets in order to distinguish malignant from nonmalignant populations (Figure 4A–C). Malignant epithelial cells were defined by broad inferred CNV gains or losses and high‐CNV inferCNV subcluster assignment relative to immune and stromal reference cells, whereas nonmalignant epithelial cells showed reference‐like CNV profiles. We then stratified epithelial cells into *CDKN1A*‐high and *CDKN1A*‐low groups using the median normalized *CDKN1A* expression level within each dataset as the cutoff (Figure 4D–F). Notably, nonmalignant epithelial cells appeared relatively enriched in the *CDKN1A*‐high group across all three cancer types. Specifically, in pancreatic cancer, approximately 61% of epithelial cells in the *CDKN1A*‐high group were classified as nonmalignant, compared with 44% in the *CDKN1A*‐low group (Figure 4G). Consistently, in lung cancer, the proportion of malignant epithelial cells was lower in the *CDKN1A*‐high group (8%) than in the *CDKN1A*‐low group (13%) (Figure 4H). A similar pattern was observed in breast cancer, where malignant epithelial cells accounted for 20.1% in the *CDKN1A*‐high group versus 31.1% in the *CDKN1A*‐low group (Figure 4I).

Subsequently, we applied the PROGENy R package to infer pathway activity differences between *CDKN1A*‐high and *CDKN1A*‐low malignant epithelial cells across pancreatic, lung, and breast cancers [44]. Pathway activity matrices were *z*‐score normalized for visualization and comparative interpretation. In pancreatic cancer, *CDKN1A*‐high malignant epithelial cells exhibited relatively elevated MAPK and JAK–STAT pathway activities, whereas PI3K and NF‐*κ*B signaling showed comparatively lower activity (Figure 4J, left). In lung cancer, *CDKN1A*‐high cells displayed increased TGF‐*β*, androgen, and p53 pathway activities, accompanied by relatively reduced PI3K‐associated signaling (Figure 4J, middle). In breast cancer, *CDKN1A*‐high cells demonstrated relatively lower estrogen‐ and androgen‐associated signaling activities compared with *CDKN1A*‐low cells (Figure 4J, right). To assess the robustness of these findings against threshold selection bias, we repeated the analysis using alternative quartile‐based stratification strategies. The inferred pathway activity differences remained highly concordant across cutoff schemes in all three cancer types (Spearman′s *R* > 0.90; Figure S6), indicating that the observed transcriptional patterns were stable and not driven by arbitrary *CDKN1A* dichotomization. Collectively, these findings indicate that *CDKN1A* expression is associated with distinct transcriptional states within malignant epithelial populations across multiple adenocarcinoma contexts.

### 3.5. Pseudotime Trajectory and Cellular Interaction Analysis

**Figure 5:**
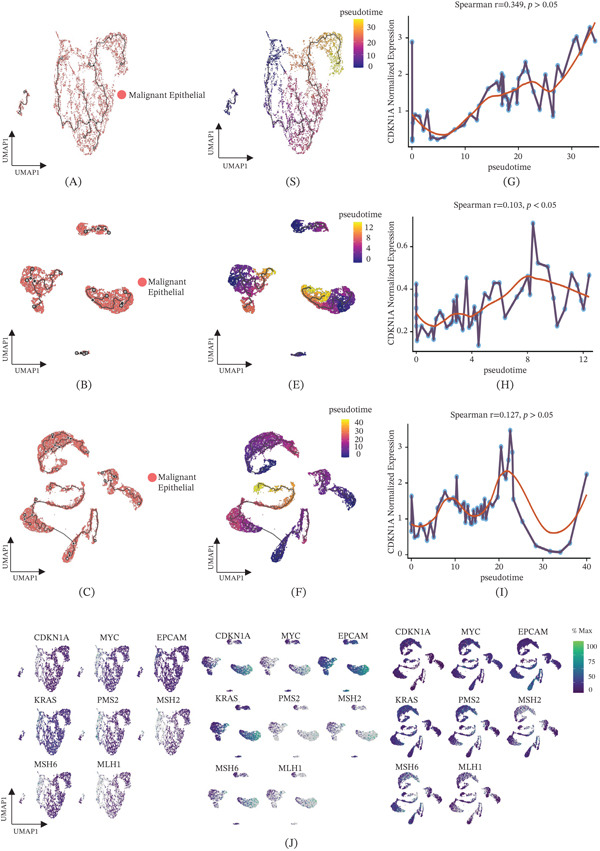
Pseudotime trajectory analysis based on *CDKN1A* expression in inferCNV‐defined malignant epithelial cells. UMAP depicting partition of malignant epithelial cells in pancreatic cancer in (A) Monocle3, (B) lung cancer, and (C) breast cancer. UMAP highlighting the inferred transcriptional continuum of malignant epithelial cells in (D) pancreatic cancer, (E) lung cancer, and (F) breast cancer. Dynamics of *CDKN1A* expression along pseudotime in (G) pancreatic cancer, (H) lung cancer, and (I) breast cancer. Each point represents the mean normalized expression of *CDKN1A* within equal pseudotime bins, and the smooth line indicates the fitted expression trend. (J) The alterations of *CDKN1A*‐related genes over pseudotime in pancreatic cancer (Left), lung cancer (Middle), and breast cancer (Right). Pseudotime denotes an inferred transcriptional continuum and should not be interpreted as direct evidence of temporal tumor progression.

Pseudotime analysis is a computational method that arranges cells along a trajectory based on their gene expression patterns, enabling the reconstruction of inferred transcriptional continua such as differentiation‐associated or activation‐associated cell‐state variation [31–33, 45]. Therefore, pseudotime analysis was performed using Monocle3 to characterize transcriptional variation within inferCNV‐defined malignant epithelial cells across pancreatic, lung, and breast cancers. To avoid confounding effects from distinct cell lineages, trajectory inference was restricted to malignant epithelial populations. Cells were ordered along a low‐dimensional manifold based on transcriptomic similarity without imposing gene expression–based priors for trajectory rooting. Given the cross‐sectional nature of these scRNA‐seq datasets, the inferred pseudotime represents a data‐driven transcriptional continuum rather than direct evidence of tumor progression, tumor evolution, or malignant transition. Across all three cancer types, *CDKN1A* expression exhibited modest but statistically significant associations with pseudotime (Spearman *r* = 0.349 in pancreatic cancer, *r* = 0.103 in lung cancer, and *r* = 0.127 in breast cancer; Figure 5A–F). Notably, both the direction and magnitude of these correlations varied across datasets, consistent with the arbitrary orientation of pseudotime and the absence of a predefined temporal axis. Binned expression analysis further demonstrated gradual variation of *CDKN1A* expression along the inferred trajectory, indicating that *CDKN1A*‐high and *CDKN1A*‐low cells occupy distinct regions within the transcriptional landscape (Figure 5G–I). To further characterize transcriptional heterogeneity, we applied CytoTRACE, which estimates cellular differentiation states based on transcriptional diversity. CytoTRACE scores exhibited substantial variability across malignant epithelial cells, suggesting the presence of heterogeneous transcriptional states (Figure S7A, S7B, S7C). *CDKN1A* expression showed a positive association with CytoTRACE scores, indicating that *CDKN1A*‐high and *CDKN1A*‐low cells are distributed across distinct regions of the inferred transcriptional state space (Figure S7D, S7E, S7F). In addition, several genes, including EPCAM, KRAS, MLH1, MSH6, MYC, and PMS2, displayed coordinated expression patterns along pseudotime (Figure 5J, Figure S8), suggesting structured transcriptional variation within malignant epithelial populations. Collectively, these findings indicate that *CDKN1A* expression is associated with a continuous transcriptional gradient in malignant epithelial cells, rather than serving as a discrete marker of tumor progression or a driver of directional state transitions.

**Figure 6:**
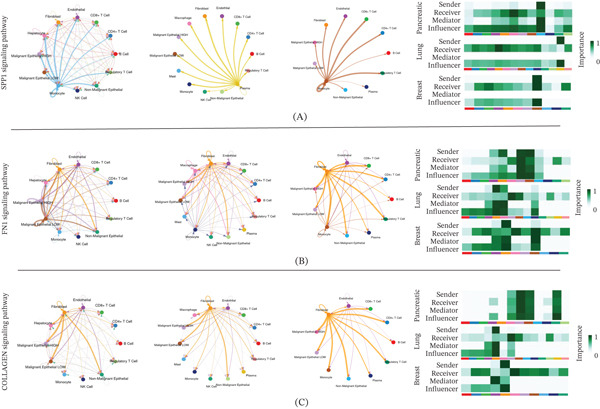
Computationally inferred ligand‐receptor interactions between malignant epithelial cell populations with different *CDKN1A* expression levels in pancreatic, lung cancers, and breast cancer. CellChat‐inferred interaction networks in selected signaling pathways, including SPP1 in (A) pancreatic cancer, (B) FN1 in lung cancer, and (C) collagen in breast cancer. Left, signal pathway network‐level visualization; Right, inferred cell roles in signal pathways.

We subsequently utilized CellChat, a computational tool developed to infer ligand‐receptor interaction networks within the TME, to compare putative signaling interactions in cells with high versus low *CDKN1A* expression in pancreatic, lung, and breast cancers [37]. High *CDKN1A* expression was predicted to be associated with stronger and more frequent ligand‐receptor interaction signals. To gain deeper insights, we examined overall signaling pathway information flow, focusing on key pathways such as SPP1, FN1, and COLLAGEN, which are involved in extracellular matrix remodeling and TME organization [46–48]. In the SPP1 signaling pathway, high *CDKN1A*‐expressing epithelial cells were inferred to act as both signal senders and receivers, suggesting an association with ligand‐receptor programs related to migration and invasion in pancreatic cancer (Figure 6A). Similarly, the FN1 and COLLAGEN pathways, well‐known regulators of tissue structure and extracellular matrix organization, were enriched in high *CDKN1A*‐expressing cells, indicating altered ECM‐related inferred signaling in lung cancer and breast cancer, respectively (Figure 6B,C) [47, 48]. Importantly, these findings are derived from computational inference based on ligand‐receptor expression patterns and should not be interpreted as direct experimental evidence of functional cell–cell communication. Rather, they represent putative intercellular signaling networks that may contribute to tumor microenvironment organization. Further validation using complementary approaches, such as spatial transcriptomics, perturbation‐based experiments, and coculture assays, will be necessary to determine whether these predicted interactions are functionally relevant within the tumor microenvironment.

### 3.6. *CDKN1A* and Tumor Therapy

**Figure 7:**
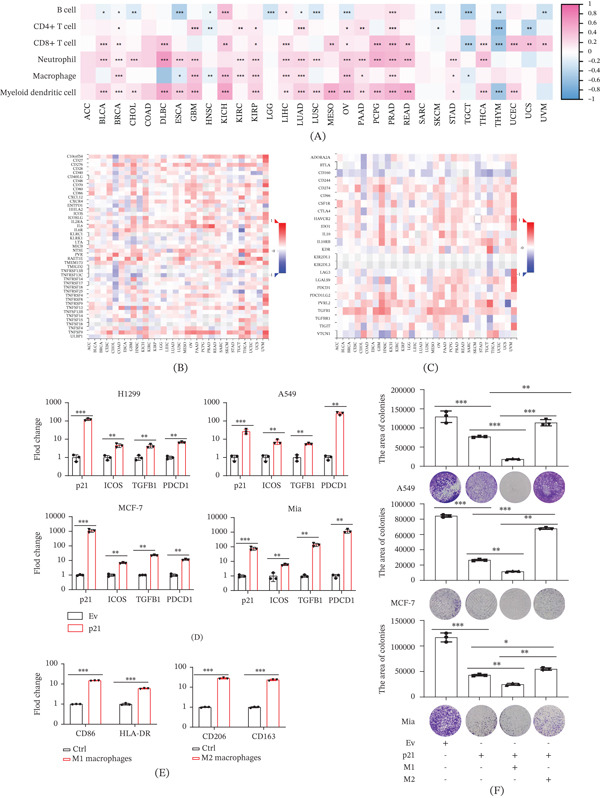
*CDKN1A*/p21 is associated with immune regulation and modulates macrophage‐mediated tumor cell responses. (A) The UCSCXenaShiny database showing the correlation between *CDKN1A* expression and the infiltration of six immune cells across various cancers. The correlation between *CDKN1A* expression and (B) immune stimulators and (C) inhibitor in cancers through TISIDB. (D) RT‐qPCR showing overexpression of p21 increases the expression of immune‐related molecules, including the immunostimulatory marker *ICOS* and immunoregulatory factors *TGFB1* and *PD-1* in multiple cancer cell lines (H1299, A549, MCF‐7, and Mia). (E) THP‐1 cells were differentiated into macrophages using PMA and further polarized into M1 macrophages by LPS + IFN − *γ* or M2 macrophages by IL‐4 treatment. Polarization efficiency was validated by RT‐qPCR analysis of M1 markers (*CD86*, *HLA-DR*) and M2 markers (*CD206*, *CD163*). (F) Cancer cells (A549, MCF‐7, and Mia) transfected with control vector or p21 were cocultured with M1 or M2 macrophage‐conditioned media. Colony formation assays were performed to assess cell proliferation. M1 macrophages enhance the inhibitory effect of p21 on tumor cell growth, whereas M2 macrophages attenuate this effect. *Note:*  ^∗^ *p* < 0.05,  ^∗∗^ *p* < 0.01,  ^∗∗∗^ *p* < 0.001 by two‐tailed Student′s *t-*test.

To investigate the immunological relevance of *CDKN1A*, we analyzed its association with immune cell infiltration using UCSCXenaShiny and TIMER [38, 49]. Our analyses showed that *CDKN1A* expression was positively associated with immune infiltrations of CD4 + T cell, CD8 + T cell, neutrophil, macrophage, and myeloid dendritic cell infiltration across diverse cancer types (Figure 7A). To further explore the broader immunomodulatory role of *CDKN1A*, we assessed its correlation with immune‐related genes using the TISIDB database [39]. *CDKN1A* was positively correlated with several immunostimulatory molecules, including *CD28*, *ICOS*, and *CD40*, which are key costimulatory receptors that promote T‐cell activation, proliferation, and cytokine secretion (Figure 7B) [50]. Conversely, *CDKN1A* was also positively associated with immunosuppressive markers, such as *PDCD1* (*PD-1*), *CTLA4*, *LAG3*, and *TGFB1*, all of which mediate T‐cell exhaustion, regulatory T‐cell induction, and immune tolerance (Figure 7C) [51, 52]. We further found that overexpression of p21 increases immune‐related molecules, including the immunostimulatory marker *ICOS* and the immunoregulatory factors *TGFB1* and *PD-1* (Figure 7D). Consistent with this, macrophage signatures and M1‐associated markers (CD80 and CD86) were positively correlated with *CDKN1A* expression (Figure 7A,B), prompting us to investigate whether macrophage polarization influences p21‐mediated cellular effects. THP‐1 cells were successfully polarized into M1 (PMA + LPS + IFN − *γ*) or M2 (PMA + IL − 4) macrophages, as confirmed by the induction of corresponding markers (Figure 7E). We then cocultured M1 or M2 macrophages with A549, MCF‐7, and Mia cells overexpressing p21 or control vectors. Notably, M1 macrophages enhanced the inhibitory effect of p21 on cell proliferation, whereas M2 macrophages attenuated this effect (Figure 7F). These dual associations suggest that *CDKN1A* functions as a context‐dependent regulator of tumor immunity, balancing immune activation and suppression in line with its dual roles in tumor suppression and immune homeostasis.

**Figure 8:**
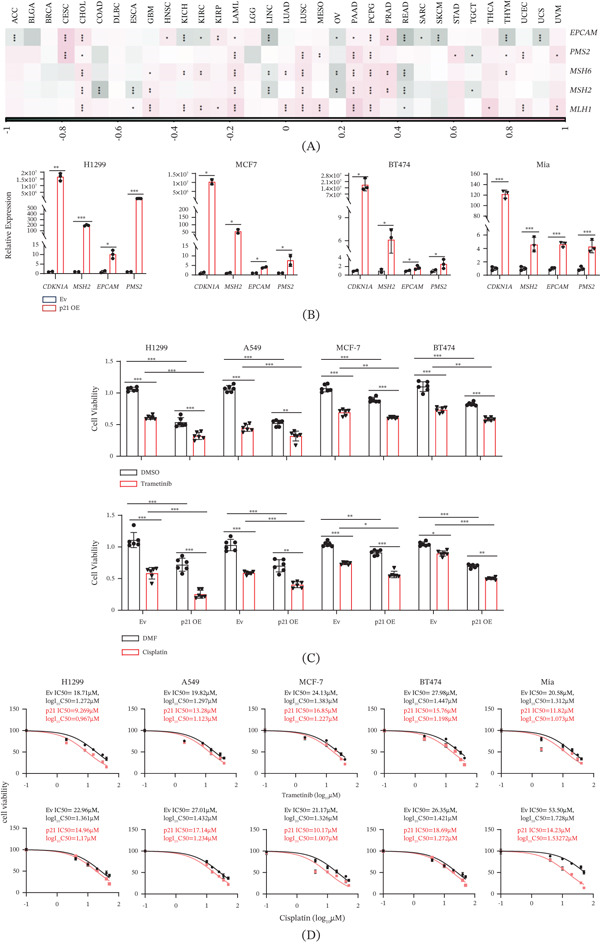
*CDKN1A* enhances cell sensitivity to multiple chemotherapeutic agents. (A) Correlation between *CDKN1A* expression levels and five MMR genes in diverse cancers. (B) RT‐qPCR showing MMR gene expression upon p21 overexpression in H1299, MCF‐7, BT474, and Mia. (C) CCK8 assay indicating overexpression of p21 enhances cell sensitivity to trametinib (5 *μ*M), cisplatin (5 *μ*M) treatment in different cell lines; *n* = 6. (D) Dose‐response curves and IC50 analyses of trametinib and cisplatin in H1299, A549, MCF‐7, BT474, and Mia cells with control or p21 overexpression. *Note:*  ^∗^ *p* < 0.05,  ^∗∗^ *p* < 0.01,  ^∗∗∗^ *p* < 0.001 by two‐tailed Student′s *t*‐test.

MMR plays a crucial role in determining how well certain cancers respond to immunotherapy, particularly in determining the response to immune checkpoint inhibitors (ICIs) [53]. In this context, we investigated the relationship between *CDKN1A* and MMR gene expression across various cancer types. Our analysis revealed that *CDKN1A* was significantly correlated with at least three out of five core MMR genes in several tumor types, including CHOL, GBM, KICH, LAML, LIHC, LUSC, OV, PAAD, PCPG, PRAD, and READ (Figure 8A). To validate these findings, we examined the mRNA levels of MMR genes following p21 overexpression. Notably, enforced p21 stimulated *MSH2*, EPCAM, and PMS2 expression in H1299, MCF‐7, BT474, and Mia cells (Figure 8B). Interestingly, these related genes exhibited distinct trends at the single‐cell level in pancreatic cancer and lung cancer (Figure 5J and Figure S8). To evaluate whether p21 alters in vitro drug sensitivity, we overexpressed p21 in multiple cells, followed by treatment with front‐line therapeutic agents, including trametinib and cisplatin. p21 overexpression increased cellular sensitivity to trametinib and cisplatin in H1299, A549, MCF‐7, and BT474 cells (Figure 8C). We further found that p21 overexpression reduced the IC50 values of trametinib and cisplatin in H1299, A549, MCF‐7, BT‐474, and Mia cells (Figure 8D). These findings suggest that p21 may modulate cellular sensitivity to targeted therapy and chemotherapy under experimental conditions.

## 4. Discussion

In this study, we provide an integrative characterization of *CDKN1A*/p21 across multiple cancer types using pancancer analyses, single‐cell transcriptomics, and functional validation. Our findings extend the canonical view of p21 as a cell cycle regulator by suggesting a broader, context‐dependent association with tumor‐related transcriptional states, TME interactions, and in vitro drug sensitivity.

As one of the best‐characterized transcriptional targets of p53, p21 plays a central role in mediating p53‐dependent tumor suppression. In early‐stage or p53‐proficient tumors, nuclear p21 restrains uncontrolled proliferation, facilitates DNA repair, and induces senescence or apoptosis, consistent with its tumor‐suppressive role [4]. For example, p21 has been identified as evolutionarily conserved core effector of the p53 network, coordinating cell cycle arrest and apoptosis across cancer contexts [54]. Conversely, loss or suppression of p21 compromises p53‐dependent tumor suppression [55]. In line with this, our functional experiments across multiple cancer cell lines demonstrate that p21 overexpression suppresses proliferation, reduces clonogenic capacity, and enhances sensitivity to chemotherapeutic agents. Notably, these effects were observed across cell lines with distinct p53 statuses, suggesting that p21 may retain partial tumor‐suppressive functions independent of canonical p53 signaling.

scRNA‐seq has fundamentally transformed our understanding of tumor biology by enabling high‐resolution dissection of the TME at the level of individual cells [14]. This technology allows the identification of distinct cellular subtypes, functional states, and lineage relationships within tumors, including malignant cells, immune infiltrates, and stromal components. Currently, the role of p21 in tumor immunity remains incompletely understood, particularly from an integrative multiomics and single‐cell perspective. Our single‐cell analyses reveal that *CDKN1A*/p21 expression is enriched in nonmalignant epithelial populations and varies across an inferred malignant epithelial transcriptional continuum, indicating a potential association with epithelial cell‐state heterogeneity. In addition, computational analyses suggest associations between *CDKN1A*/p21 and extracellular matrix‐related inferred ligand‐receptor signaling pathways, as well as immune‐related features, including immune cell infiltration and the expression of immunomodulatory molecules. Previous studies reported that p21 affects tumor immunity. For example, p21 supports Langerhans cell survival and promotes regulatory T‐cell generation after irradiation, highlighting its dual role in immune homeostasis and radiation‐induced immunosuppression [15]. p21 maintains CD4^+^ T‐cell activity and prevents exhaustion, thereby sustaining effective antitumor immune responses in colorectal cancer [16]. p21 also enhances macrophage‐mediated clearance of leukemia cells by repressing SIRP*α* and promoting prophagocytic reprogramming, offering a novel therapeutic strategy for acute leukemia [56]. In our study, we conducted coculture experiments to provide functional insight into the interaction between p21‐expressing tumor cells and macrophages. Our results demonstrate that M1 macrophages enhance, whereas M2 macrophages attenuate the inhibitory effect of p21 on tumor cell proliferation. We further show that p21 regulates immune‐related molecules, including *ICOS*, *TGFB1*, and *PD-1*. These findings provide functional evidence supporting a context‐dependent role of p21 in tumor‐immune interactions and suggest that the impact of p21 on tumor behavior may be modulated by the immune microenvironment. Nevertheless, the underlying molecular mechanisms remain to be elucidated, and further studies using immune‐competent models will be required.

In addition, we observed that *CDKN1A* expression is associated with drug sensitivity across multiple datasets, and p21 overexpression reduced IC50 values for targeted therapies and chemotherapy in vitro. However, these findings should be interpreted with caution, as potential confounding factors such as tumor purity, tumor stage, and cellular heterogeneity were not fully controlled due to limited clinical annotation. Furthermore, prognostic association should be distinguished from treatment‐response prediction. Although our data support an association between *CDKN1A*/p21 expression and therapeutic sensitivity‐related features, they do not establish predictive utility for clinical treatment response. Therefore, the potential role of p21 as a predictive biomarker, particularly in the context of immunotherapy, remains preliminary and warrants further validation in well‐annotated patient cohorts with treatment‐specific response data and appropriate multivariable analyses.

In summary, our study highlights a multifaceted and context‐dependent role of *CDKN1A*/p21 in cancer biology, linking tumor cell‐intrinsic regulation with microenvironmental interactions and in vitro therapeutic sensitivity‐associated phenotypes. Notably, this study represents, to our knowledge, the first systematic characterization of *CDKN1A* using scRNA‐seq, providing new insights into its cell‐type‐specific functions within the TME. Further mechanistic and clinical studies will be essential to fully define the role of *CDKN1A*/p21 as a biomarker and therapeutic target.

## 5. Limitations and Future Directions

This study has several limitations. First, although we integrated multiple public datasets and experimental validations, the correlation analyses between *CDKN1A* expression and drug sensitivity may be influenced by unaccounted confounding factors, such as tumor purity, tumor stage, and cellular composition, which were not fully adjustable due to limited clinical annotations. Second, the associations between *CDKN1A* and immune infiltration, immune‐related molecules, and MMR pathways are largely based on computational and correlation‐based analyses, and therefore do not establish causality. Although preliminary in vitro experiments were performed, more rigorous mechanistic and in vivo studies are needed to validate these findings. Third, despite suggesting a potential role for *CDKN1A* in predicting immunotherapy response, the absence of clinical cohort data limits the translational applicability of our conclusions. Future studies incorporating well‐annotated patient cohorts and functional validation in physiologically relevant models will be essential to address these limitations.

## 6. Conclusion

In summary, this study provides an integrative multiomics and single‐cell characterization of *CDKN1A*/p21 across multiple cancer types, revealing its context‐dependent associations with tumor‐related transcriptional states, cellular differentiation‐associated features, and in vitro drug sensitivity. Our findings extend the conventional view of p21 as a cell cycle regulator by suggesting its broader involvement in TME interactions and drug sensitivity. However, given that several conclusions are based on correlative analyses, computational inference, and limited experimental validation, the clinical and mechanistic implications of *CDKN1A*/p21 should be interpreted cautiously. Further studies incorporating well‐controlled clinical datasets, treatment‐specific response endpoints, and in‐depth functional experiments will be required to establish its role as a reliable biomarker and therapeutic target.

## Author Contributions

All authors made a significant contribution to the work reported, whether that is in the conception, study design, execution, acquisition of data, analysis and interpretation, or in all these areas; took part in drafting, revising or critically reviewing the article; have agreed on the journal to which the article has been submitted; and agree to be accountable for all aspects of the work. Wenyang Zhang, Qinglong Ma, Honghui Zhao, Wenrun Li, and Zidong Feng contributed equally to this article.

## Funding

This work is supported by the Fundamental Research Funds for the Central Universities, lzujbky‐2024‐oy04, lzujbky‐2024‐it64, 561225006, 20250020160; the NHC Key Laboratory Open Fund, 23GSSYA‐11; the National Natural Science Foundation of China, 10.13039/501100001809, 82273001, 82573821; the Gansu Province Department of Science and Technology Key R&D Fund, 23YFWA0008; the Central Double First‐Class Universities Construction Fund of Lanzhou University, 561121202; the Veterinary Etiological Biology State Key Laboratory 2023 Open Fund, SKLVEB‐KFKT‐06; the Medical Innovation and Development Project of Lanzhou University, lzuyxcx‐2022‐163; and the National College Students Innovation and Entrepreneurship Training Program, 10.13039/501100013254, 202310730216.

## Disclosure

All authors have read the manuscript and gave final approval of the version to be published. Code related to the analyses in this study can be found on GitHub: https://github.com/Snnmmk/CDKN1A. A preprint version of this manuscript has previously been published on bioRxiv (Zhang et al., 2024) [56].

## Conflicts of Interest

The authors declare no conflicts of interest.

## Supporting information


**Supporting Information** Additional supporting information can be found online in the Supporting Information section. Figure S1: *CDKN1A* expression in different pathological stages of cancers. (A) IHC from the Human Protein Atlas showing p21 protein level in colon cancer, breast cancer and prostate cancer. The expression level of *CDKN1A* in different tumor stage of (B) BLCA, (C) BRCA, (D) COAD, (E) LUAD, (F) LUSC, (G) KICH, (H) READ, (I) CHOL, (J) HNSC, (K) KIRC, (L) KIRP, (M) THCA. *Note:*  ^∗^ *p* < 0.05,  ^∗∗^ *p* < 0.01,  ^∗∗∗^ *p* < 0.001. Figure S2: Gene signature analysis of *CDKN1A* low and high dataset among BLCA, COAD, LUAD and LUSC. (A) Venn diagram showing common gene signatures among different cancer types. (B) Enforced MYC represses p21 level in H1299, BT474, ZR751 and Mia cells. (C) Immunofluorescence staining indicating overexpressed MYC represses p21 level in multiple cell lines. Bubble plots showing GO analyses of *CDKN1A* Low and High datasets in (D) LUSC, (E) COAD, (F) BLCA, (G) and LUAD. Figure S3: The functional relevance of *CDKN1A* across different cancers from CancerSEA. (A) Correlations between *CDKN1A* and biological activities in different cancers. (B) Functional relevance of *CDKN1A* in LUAD. Red plots suggesting positive correlations while blue plots indicating negative correlations. (C) *CDKN1A* is correlated with metastasis, differentiation and quiescence, DNA repair and cell cycle in LUAD. *Note:*  ^∗^ *p* < 0.05,  ^∗∗^ *p* < 0.01 by two‐tailed Student′s *t-*test. Figure S4: p21 regulates gene expression. RT‐qPCR showing dysregulated genes after p21 overexpression in (A) H1299 and (B) A549.  ^∗^ *p* < 0.05,  ^∗∗^ *p* < 0.01,  ^∗∗∗^ *p* < 0.001 by two‐tailed Student′s *t-*test. Abbreviation: N.S., not significant. Figure S5: Representative Annexin‐V plots indicating apoptosis distribution in (A) H1299, (B) A549, (C) MCF‐7, (D) BT474, (E) Mia, (F) PDC0034, and (G) indicated quantifications upon p21 overexpression. *Note:*  ^∗∗^ *p* < 0.01,  ^∗∗∗^ *p* < 0.001 by two‐tailed Student′s *t-*test. Figure S6: Robustness analysis under different *CDKN1A* cutoff strategies. (A) Robustness tests performed using alternative *CDKN1A* stratification cutoff schemes, including median‐based and quantile‐based grouping methods. Consistent transcriptional patterns and pathway activity trends were observed across different cutoff strategies, supporting the robustness of the *CDKN1A*‐associated analyses and indicating that the findings were not driven by arbitrary threshold selection. Figure S7: CytoTRACE shows transcriptional heterogeneity. UMAP projections visualizing the predicted differentiation potency of single cells in (A) pancreatic cancer, (B) lung cancer, and (C) breast cancer. Cells are colored by CytoTRACE potency scores, ranging from differentiated (blue) to multipotent (red/orange). The increasing prevalence of *CDKN1A*‐high cells along the differentiation hierarchy in (D) pancreatic cancer, (E) lung cancer, and (F) breast cancer. Data show the proportion of *CDKN1A*‐high cells within each potency category, with error bars representing the 95% confidence interval. Comparison of CytoTRACE potency scores between *CDKN1A*‐high and *CDKN1A*‐low subpopulations in (G) pancreatic cancer, (H) lung cancer, and (I) breast cancer. *Note:*  ^∗∗∗^ *p* < 0.001 by two‐tailed Student′s *t-*test. Figure S8: Pseudotime trajectory analysis showing alterations of *CDKN1A* and related genes across the inferred malignant epithelial transcriptional continuum in (A) pancreatic cancer, (B) lung cancer, and (C) breast cancer.

## Data Availability

The datasets used to support the results of this study are available from the corresponding author upon request (Lei Shi, leishi@lzu.edu.cn). scRNA‐seq and TCGA data are publicly available from the cBioPortal and GEO dataset with access code described in the Materials and methods section.
