# Supplementary material for: Single‐Cell and Multiomics Characterization of p21 in Cancer Progression and Therapeutic Sensitivity
Source: Hum Mutat. 2026 Jul 6;2026:5598948. doi: 10.1155/humu/5598948 (PMC13338575; doi:10.1155/humu/5598948)
Supplement: Supplementary file 1 — Supporting Information Additional supporting information can be found online in the Supporting Information section. Figure S1: CDKN1A expression in different pathological stages of cancers. (A) IHC from the Human Protein Atlas showing p21 protein level in colon cancer, breast cancer and prostate cancer. The expression level of CDKN1A in different tumor stage of (B) BLCA, (C) BRCA, (D) COAD, (E) LUAD, (F) LUSC, (G) KICH, (H) READ, (I) CHOL, (J) HNSC, (K) KIRC, (L) KIRP, (M) THCA. Note: ∗ p < 0.05, ∗∗ p < 0.01, ∗∗∗ p < 0.001. Figure S2: Gene signature analysis of CDKN1A low and high dataset among BLCA, COAD, LUAD and LUSC. (A) Venn diagram showing common gene signatures among different cancer types. (B) Enforced MYC represses p21 level in H1299, BT474, ZR751 and Mia cells. (C) Immunofluorescence staining indicating overexpressed MYC represses p21 level in multiple cell lines. Bubble plots showing GO analyses of CDKN1A Low and High datasets in (D) LUSC, (E) COAD, (F) BLCA, (G) and LUAD. Figure S3: The functional relevance of CDKN1A across different cancers from CancerSEA. (A) Correlations between CDKN1A and biological activities in different cancers. (B) Functional relevance of CDKN1A in LUAD. Red plots suggesting positive correlations while blue plots indicating negative correlations. (C) CDKN1A is correlated with metastasis, differentiation and quiescence, DNA repair and cell cycle in LUAD. Note: ∗ p < 0.05, ∗∗ p < 0.01 by two‐tailed Student′s t-test. Figure S4: p21 regulates gene expression. RT‐qPCR showing dysregulated genes after p21 overexpression in (A) H1299 and (B) A549. ∗ p < 0.05, ∗∗ p < 0.01, ∗∗∗ p < 0.001 by two‐tailed Student′s t-test. Abbreviation: N.S., not significant. Figure S5: Representative Annexin‐V plots indicating apoptosis distribution in (A) H1299, (B) A549, (C) MCF‐7, (D) BT474, (E) Mia, (F) PDC0034, and (G) indicated quantifications upon p21 overexpression. Note: ∗∗ p < 0.01, ∗∗∗ p < 0.001 by two‐tailed Student′s t-test. Figure S6: Robustnes [file HUMU-2026-5598948-s001.pdf]

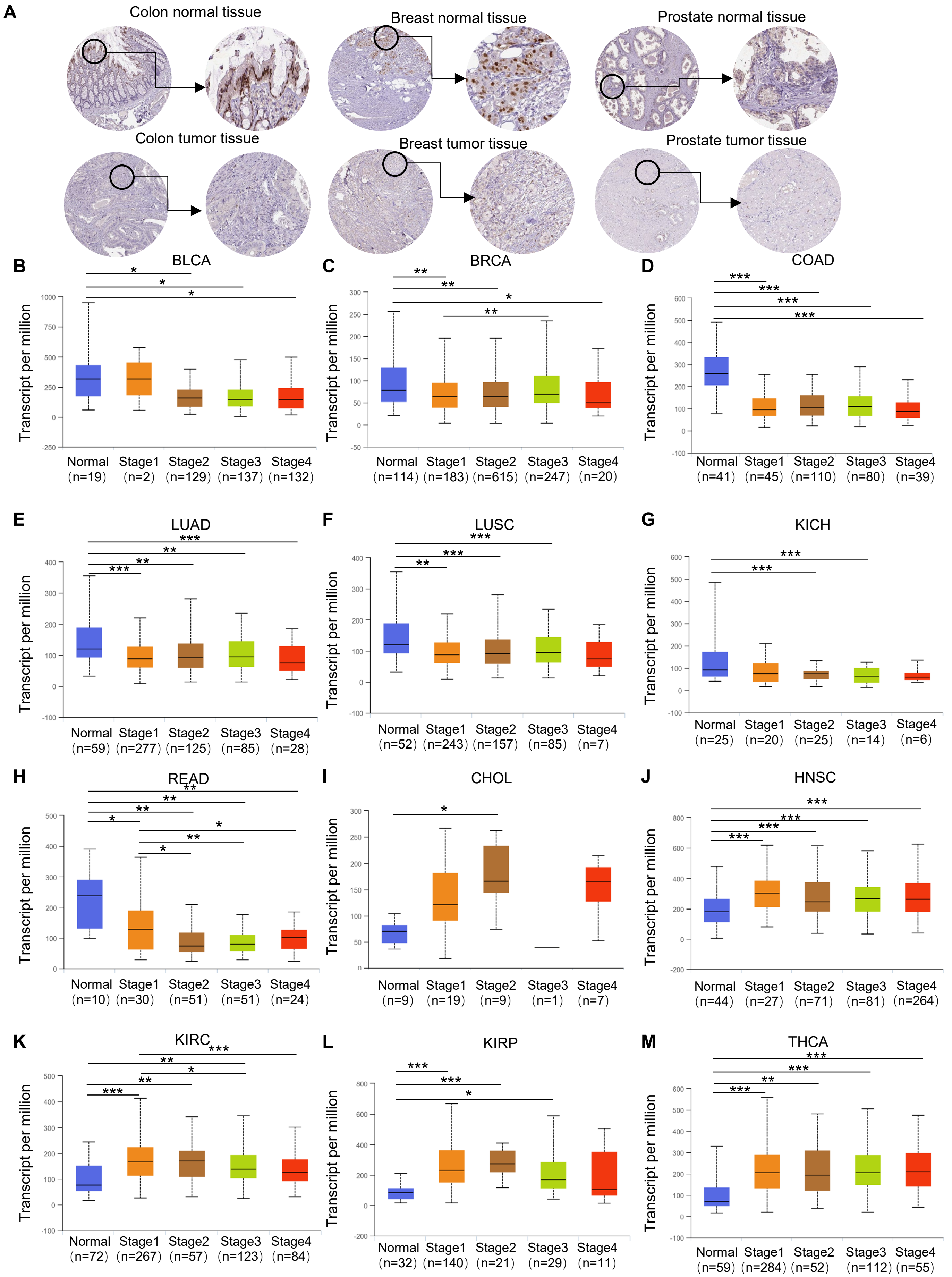

Supplementary Figure 1

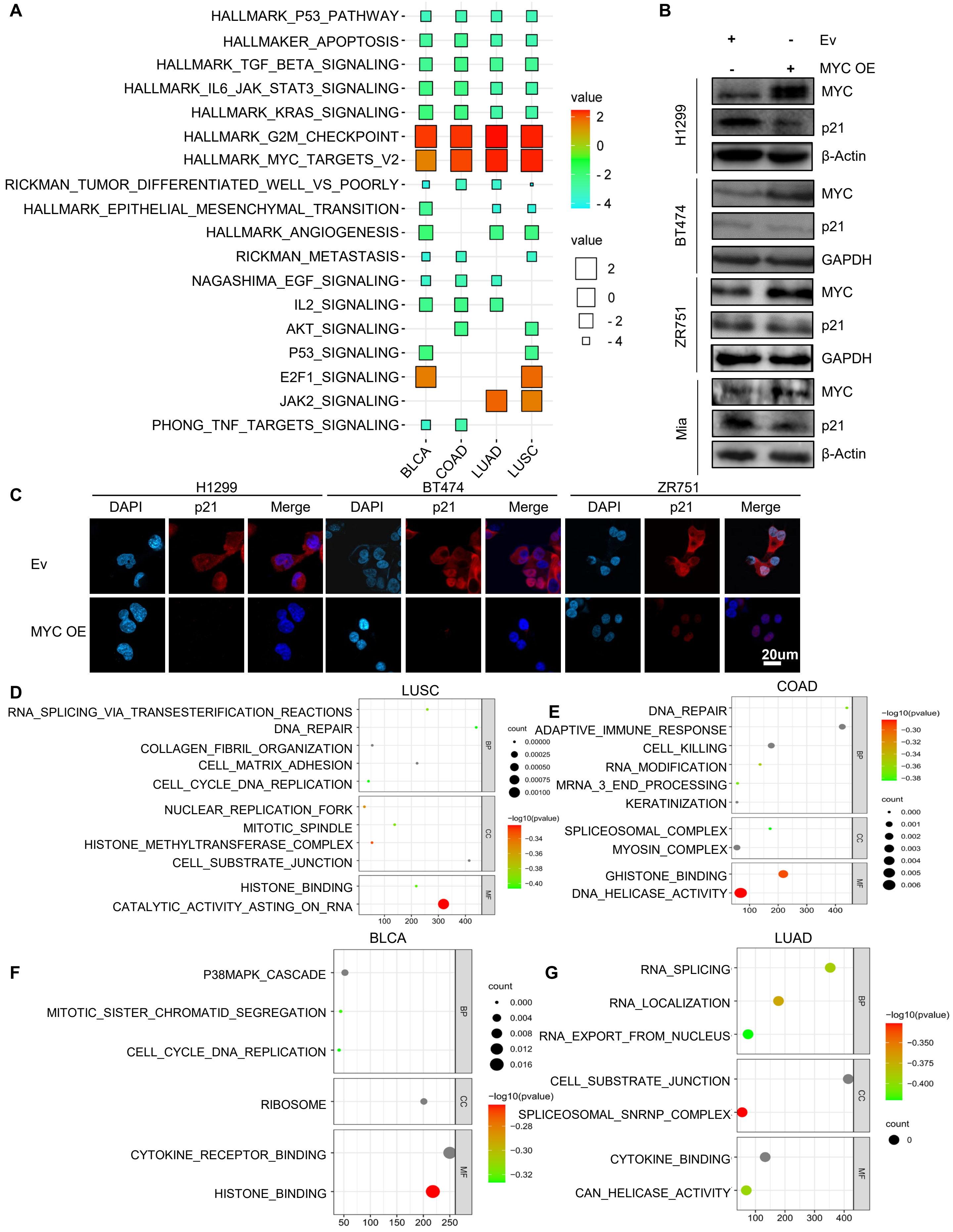

Supplementary Figure 2

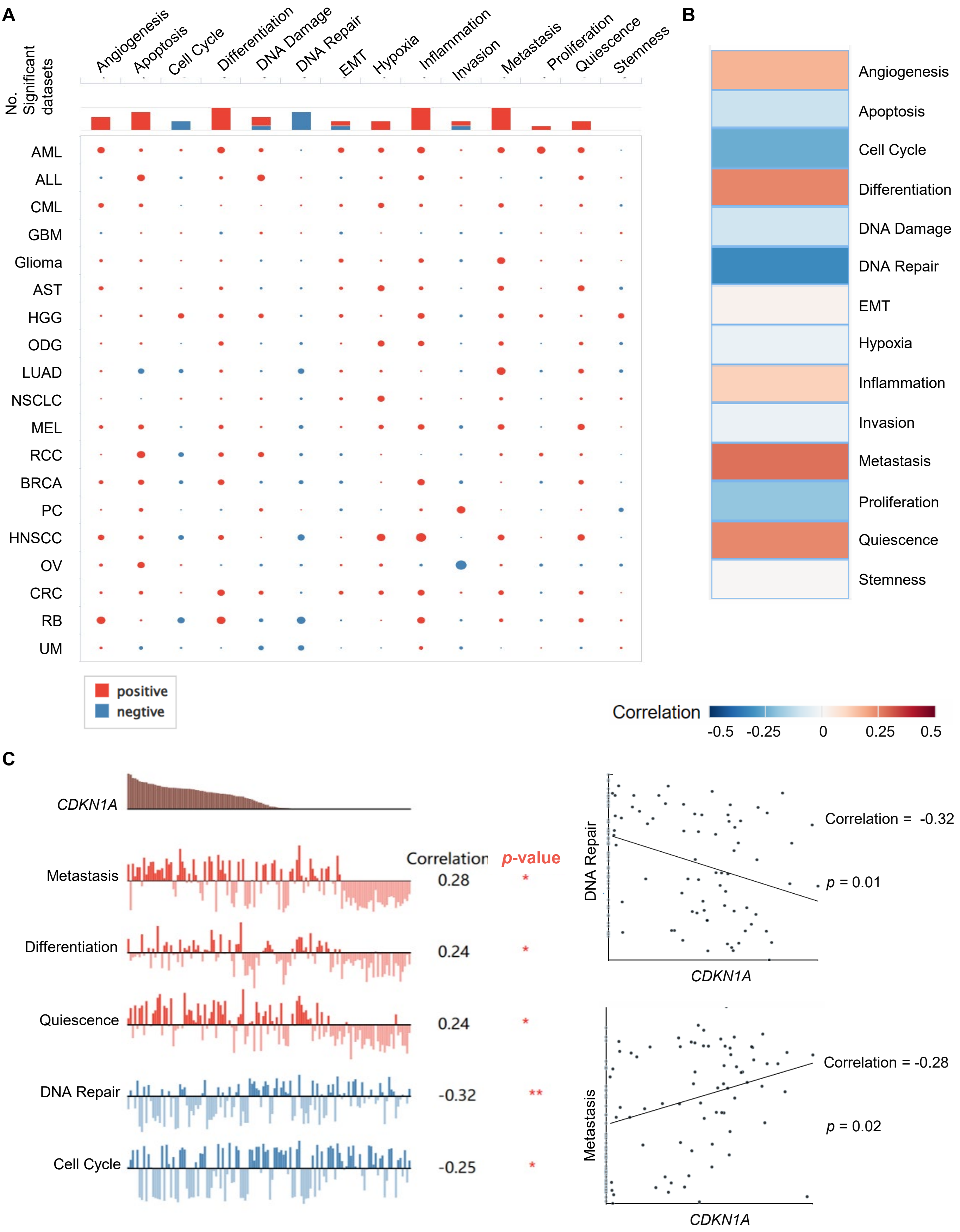

Supplementary Figure 3

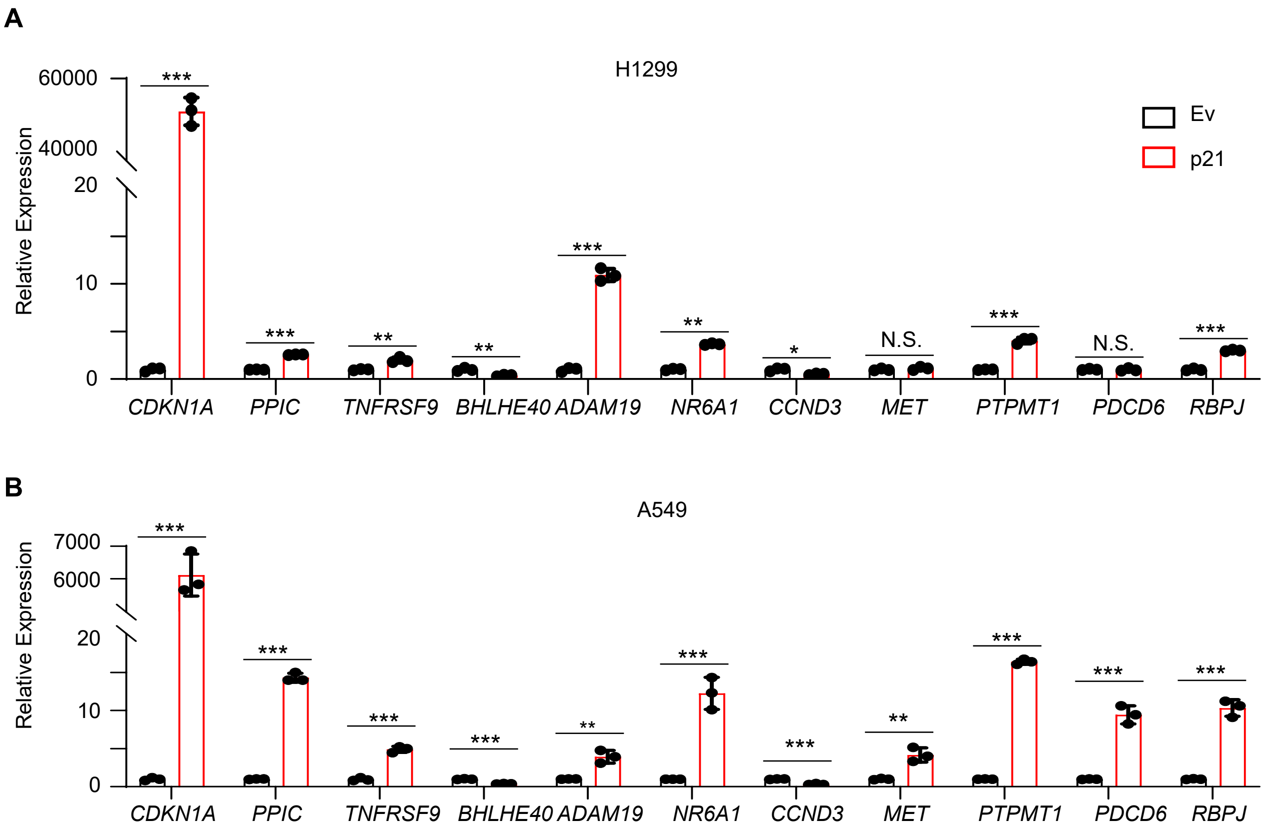

Supplementary Figure 4

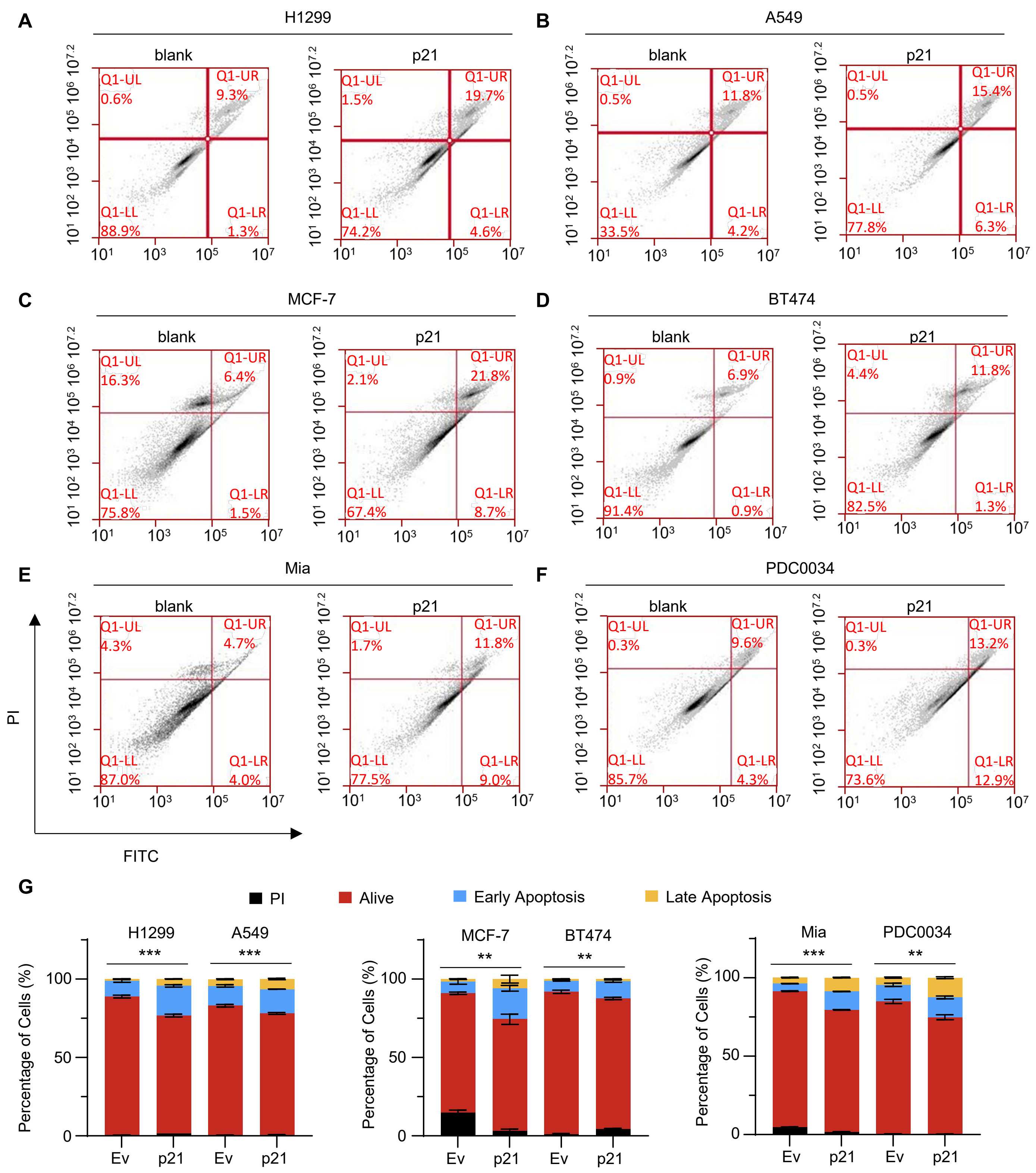

Supplementary Figure 5

A

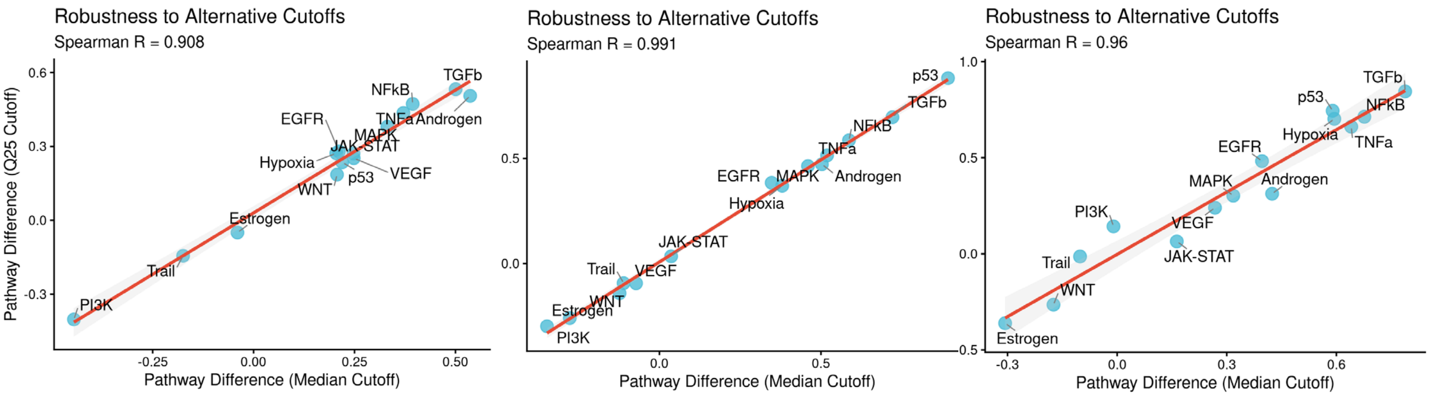

Supplementary Figure 6

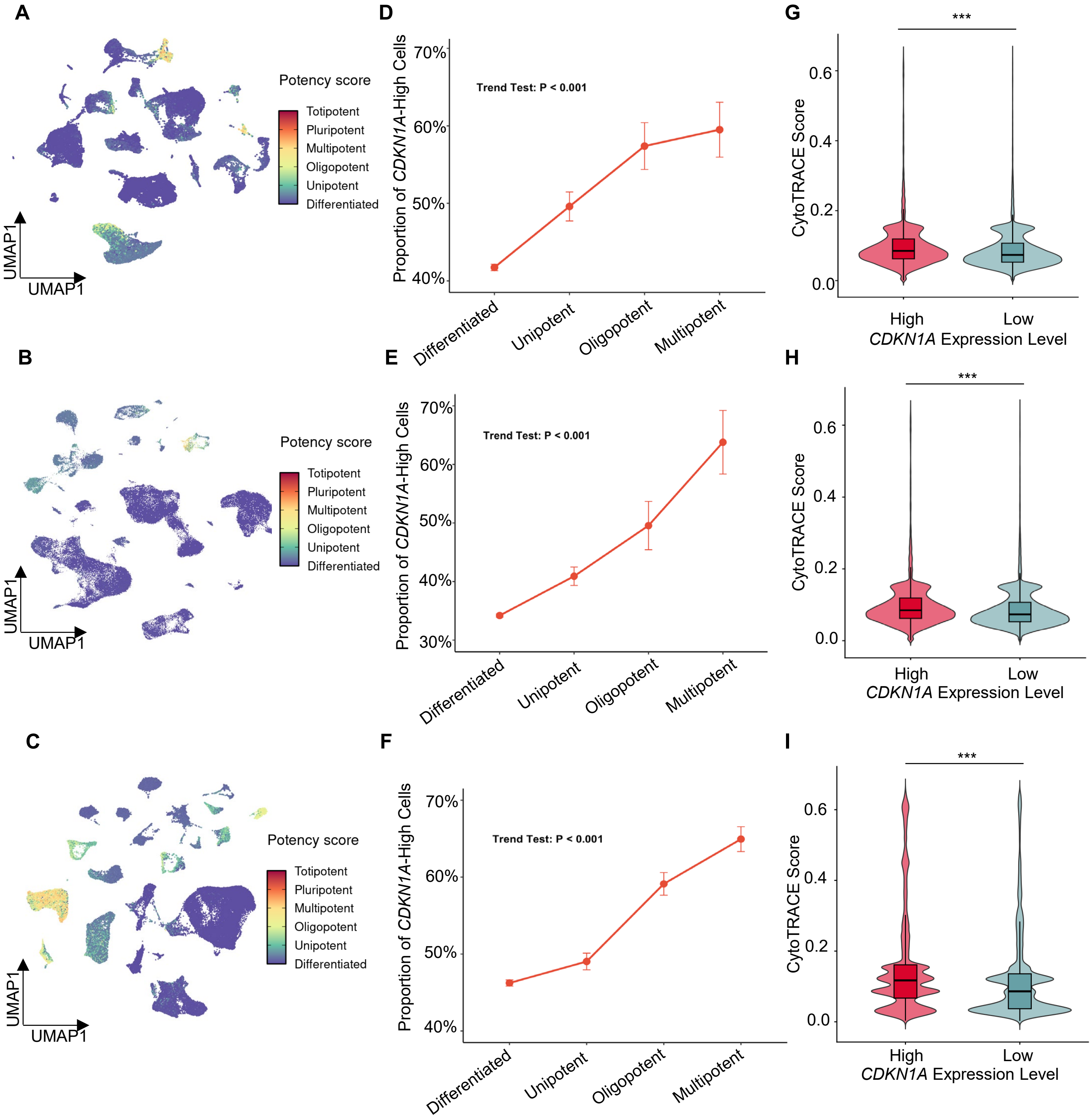

Supplementary Figure 7

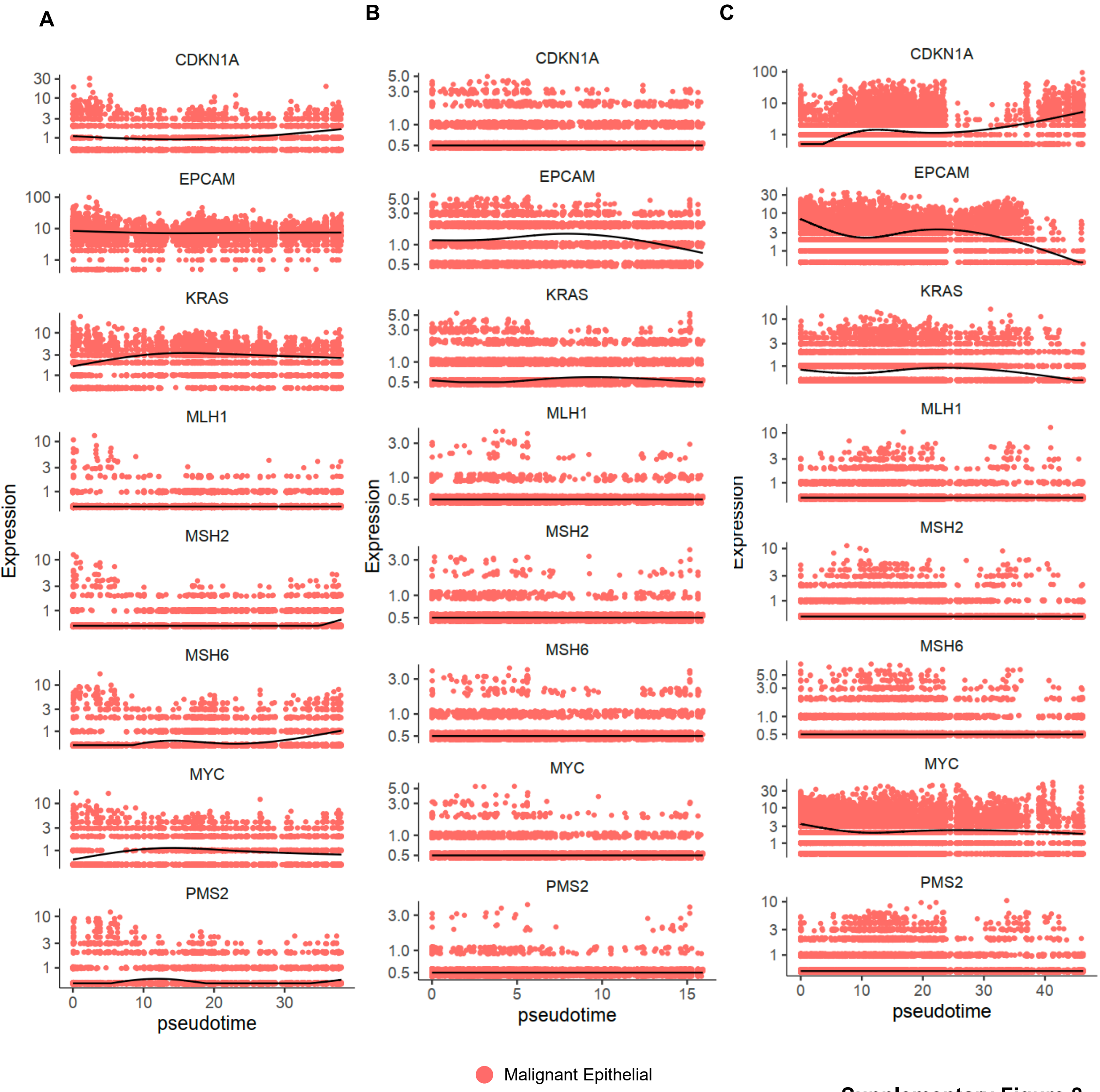

Supplementary Figure 8
